# Exosomal microRNAs in giant panda (*Ailuropoda melanoleuca*) breast milk: potential maternal regulators for the development of newborn cubs

**DOI:** 10.1038/s41598-017-03707-8

**Published:** 2017-06-14

**Authors:** Jideng Ma, Chengdong Wang, Keren Long, Hemin Zhang, Jinwei Zhang, Long Jin, Qianzi Tang, Anan Jiang, Xun Wang, Shilin Tian, Li Chen, Dafang He, Desheng Li, Shan Huang, Zhi Jiang, Mingzhou Li

**Affiliations:** 1 Institute of Animal Genetics & Breeding, College of Animal Science & Technology, Sichuan Agricultural University, Wen’jiang, 611130 China; 2 China Conservation and Research Center for the Giant Panda, Wolong, Sichuan China; 3 Novogene Bioinformatics Institute, Beijing, 100089 China

## Abstract

The physiological role of miRNAs is widely understood to include fine-tuning the post-transcriptional regulation of a wide array of biological processes. Extensive studies have indicated that exosomal miRNAs in the bodily fluids of various organisms can be transferred between living cells for the delivery of gene silencing signals. Here, we illustrated the expression characteristics of exosomal miRNAs in giant panda breast milk during distinct lactation periods and highlighted the enrichment of immune- and development-related endogenous miRNAs in colostral and mature giant panda milk. These miRNAs are stable, even under certain harsh conditions, via the protection of extracellular vesicles. These findings indicate that breast milk may facilitate the dietary intake of maternal miRNAs by infants for the regulation of postnatal development. We also detected exogenous plant miRNAs from the primary food source of the giant panda (bamboo) in the exosomes of giant panda breast milk that were associated with regulatory roles in basic metabolism and neuron development. This result suggested that dietary plant miRNAs are absorbed by host cells and subsequently secreted into bodily fluids as potential cross-kingdom regulators. In conclusion, exosomal miRNAs in giant panda breast milk may be crucial maternal regulators for the development of intrinsic ‘slink’ newborn cubs.

## Introduction

**Figure 1:**
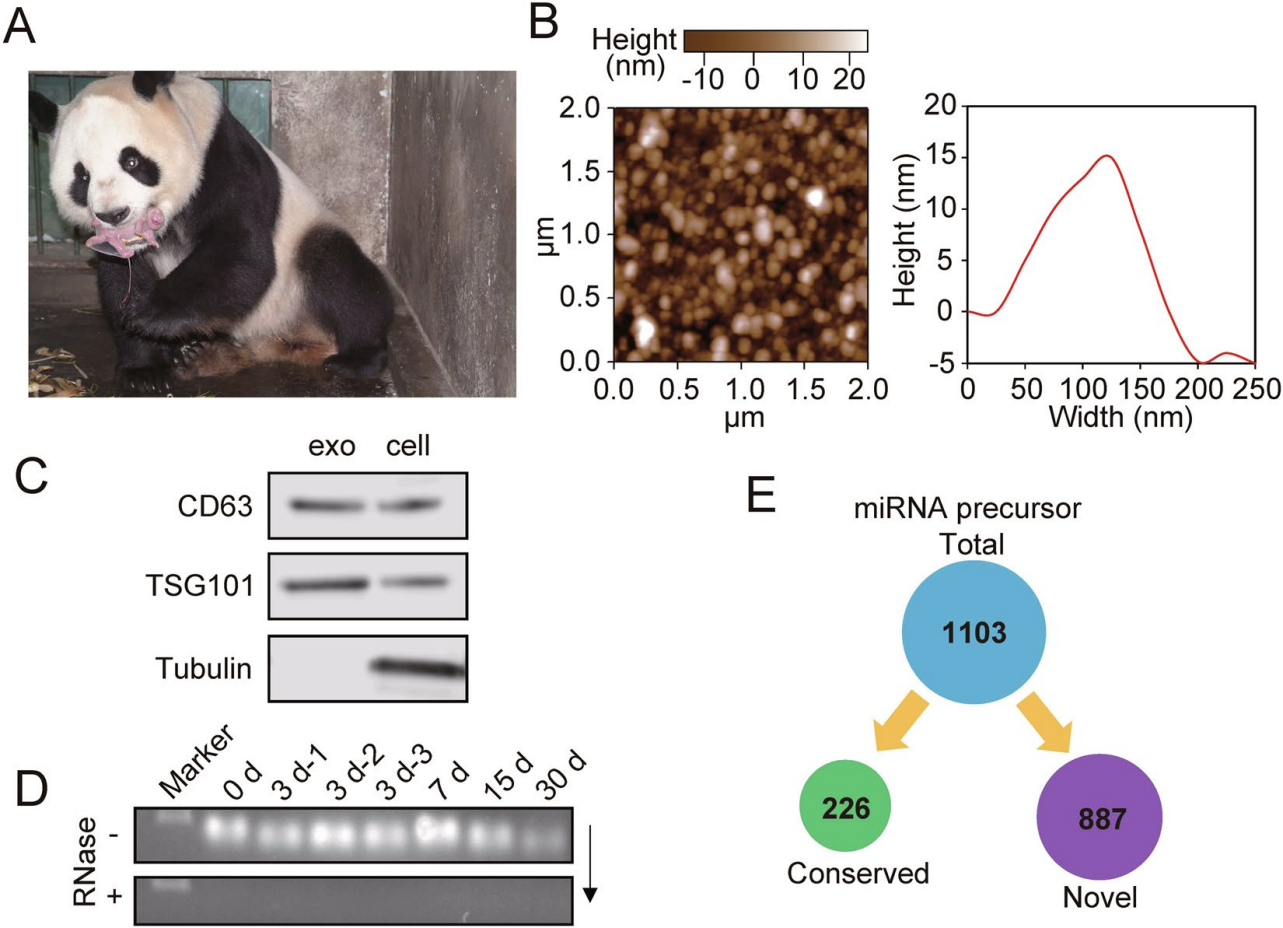
Identification of exosome miRNA in Giant Panda breast milk. (**A**) Visualization of the small neonate-maternal weight ratio of the giant panda. This image was taken by Chengdong Wang. (**B**) The particle size distribution of the milk exosomes detected using AFM. (**C**) Western blot analysis showing the enrichment of CD63 and TSG101, and the absence of Tubulin in exosomes (exo), when compared to the cellular fraction lysate of the breast milk (cell). (**D**) Total RNA was extracted from milk exosomes, and Marker represents the 100 nt marker band. The black arrow indicates the direction of electrophoresis. (**E**) Classification of giant panda miRNA precursors identified in the present study.

The giant panda (*Ailuropoda melanoleuca*) is a rare and endangered mammal and a well-known and iconic conservation species inhabiting the bamboo forests of China^1^. The giant panda has evolved several unusual biological and behavioral traits, including a famously restricted diet primarily comprising bamboo, an extremely low fecundity rate, and an enlarged radial sesamoid that functions as a thumb^2^. Giant pandas not only have the smallest neonate-maternal weight ratio (1/900) among eutherian mammals (Fig. 1A) but also exhibit the longest gestation. The adult giant panda has a body length of 120–150 cm and weighs 75–160 kg, whereas the newborn cub is extremely altricial with a body length of approximately 15 cm and a weight of 90–130 g^3^. The average daily weight gain of the giant panda within the first four months reaches about 70 g^4^. Taken together, the small neonate-maternal weight ratio and high growth rate of the newborn cubs indicates a large requirement of nutrition at the early postnatal stage that is primarily provided by the breast milk^5^.

Breast milk is the ideal source of nutrition for mammalian infants^6^ and contains a balance of nutrients that meets the infant requirements for brain development and growth, providing significant health benefits compared to formula^7^. These benefits include a decreased risk of diseases such as meningitis, respiratory infections, sudden infant death syndrome, diabetes, cancer and obesity. The mechanisms underlying these beneficial effects are poorly characterized but may be influenced through macromolecules, including growth factors, antibodies, and small nucleic acids^8, 9^.

MicroRNAs (miRNAs), a family of small non-coding RNAs 20–24 nucleotides (nt) in length, are evolutionarily conserved mediators of post-transcriptional gene regulation in eukaryotes [10]. They are ubiquitously expressed in mammalian solid tissues^10, 11^, and recently, extracellular miRNAs have been isolated from various bodily fluids (serum, saliva, urine and breast milk)^12–15^ and associated with various pathological conditions^16^. These circulating miRNAs are primarily delivered by exosomes, which are membranous vesicles released by various cell types through exocytosis^17^. Recent reports indicates that exosome-loaded miRNAs are highly enriched in breast milk of humans^8, 18^, cows^19^, pigs^20^, and exhibited a high stability to harsh conditions, like high temperature and low pH. These results also indicate that small RNAs transferred by exosomes in breast milk play an important role in the postnatal development of infants. Unfortunately, to date, the miRNA species of the giant panda have not been identified, and the potential functions of breast milk-derived exosomal miRNAs of the giant panda remain unknown.

Thus, we hypothesize that exosomal miRNAs are enriched in giant panda breast milk and modulate the physiology, immunity, and feeding behavior of newborn cubs. To test this hypothesis, we measured the miRNA expression profiles in giant panda milk exosomes across five lactation stages (0, 3, 7, 15 and 30 days after delivery, hereafter referred to as 0 d, 3 d, 7 d, 15 d and 30 d, respectively).

## Results and Discussion

### Identification of endogenous exosomal miRNAs in giant panda breast milk

Exosomes were isolated from giant panda breast milk by ultracentrifugation and were investigated using atomic force microscope (AFM) at the nanometer scale. The results are indicative of that giant panda breast milk contains a large number of round or oval membrane vesicles, which had an approximate width of 130 nm and a height of 15 nm (Fig. 1B); they exhibited classic morphological ultrastructure similar to the exosomes derived from human saliva, urine and *in vitro* cells^21–25^. According to the recommendation of the International Society for Extracellular Vesicles (ISEV)^26^, the identity of milk-derived exosomes were also confirmed by Western blot analysis, which showing expected selective enrichment of exosomal protein markers CD63 and TSG101, and the absence of Tubulin in exosomes compared to the cellular fraction lysate of the breast milk (Fig. 1C). We also observed that the milk exosomes contained a considerable number of RNA sequences shorter than 100 nt in length (Fig. 1D and Supplementary Fig. S1), confirming the enrichment of miRNA-loaded exosomes in the breast milk of the giant panda.

We then used small RNA-seq to reveal the miRNA species and abundance in seven small RNA libraries isolated from the exosomes of five lactation periods. Each small RNA library generated ~9.50 million (M) (9.50 ± 1.31 M, *n* = 7) raw reads, resulting in more than 66.47 M raw reads for seven libraries. On average, 65% (65.55 ± 11.94%, *n* = 7) of the raw reads in each library passed the quality filter and were defined as reliable miRNA candidates (Supplementary Fig. S2A), consistent with the canonical size range of mammalian miRNAs (Supplementary Fig. S2B), confirming the presence of miRNAs in the exosomes of giant panda milk. After mapping to the giant panda genome (*Ailuropoda melanoleuca*, AilMel1.0) and the known miRNA precursors (pre-miRNAs) of 24 mammals deposited in miRBase 21.0, a total of 1,103 pre-miRNAs encoding 1,236 mature miRNAs were identified in the seven libraries, including 320 conserved and 916 novel miRNAs (lineage specific) (Fig. 1E and Supplementary Table S1). There were distinct pre-miRNAs encoding identical mature miRNAs, resulting in 1,236 mature miRNAs corresponding to 1,191 unique miRNA sequences (Supplementary Table S2). The highly correlated biological replicates had similar frequency distributions for global miRNA expression (mean Pearson’s *r* = 0.84) (Supplementary Fig. S2C), suggesting the experimental reliability of the small RNA-sequencing data obtained in the present study. In addition, the clone sequencing and qRT-PCR analysis of 14 randomly selected miRNAs further confirmed the small RNA-seq results (Supplementary Fig. S3).

### Development- and immune-related miRNAs were enriched in giant panda breast milk

**Figure 2:**
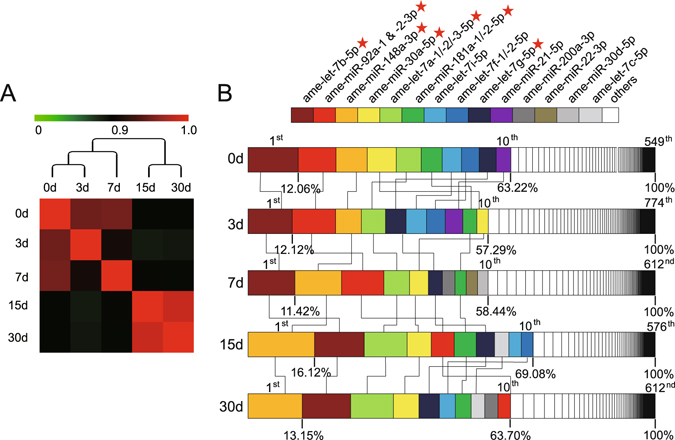
Global expression features of giant panda miRNAs. (**A**) Hierarchical clustering and heat map matrix of pairwise Spearman correlations of the counts of unique miRNAs between five lactation stages. (**B**) Top 10 unique miRNAs with the highest expression levels across five lactation stages. The seven miRNAs with the highest abundance in all five libraries are indicated with red stars.

To investigate the potential function of endogenous exosomal miRNAs, their global expression patterns were analyzed via hierarchical clustering. The miRNA expression profiles showed an obvious lactation-specific pattern. Two major branches were defined: one representing 0, 3 and 7 days and one representing 15 and 30 days (Fig. 2A). This clustering pattern corresponded to the intrinsic differences of biochemical (e.g. vitamin, sugar, sodium and creatinine) and bioactive components (e.g. Immunoglobulin, interleukin and chemokine) between the giant panda colostrum (0 to 7 days after delivery) and mature milk (8 days after delivery)^27^, implying similar regulatory roles of exosomal miRNAs as potential functional ingredients in giant panda milk. Notably, we observed that the majority of abundant miRNAs were from a few miRNA species. As shown in Fig. 2B, the top 10 unique miRNAs with the highest expression levels accounted for more than 57.29% (57.29% to 69.08%) of the total read counts of all miRNAs for each library. The unified set of the top 10 unique miRNAs over the five lactation stages corresponded to 14 unique miRNAs, including six members of the let-7 family (i.e., ame-let-7a-1/-2/-3-5p, ame-let-7b-5p, ame-let-7c-5p, ame-let-7f-1/2-5p, ame-let-7g-5p and ame-let-7i-5p). In addition, seven of these top-ranked unique miRNAs (ame-let-7b-5p, ame-miR-92a-1/-2-3p, ame-miR-148a-3p, ame-miR-30a-5p, ame-let-7a-1/-2/-3-5p, ame-miR-181a-1/-2-5p and ame-let-7g-5p) were shared by all five lactation stages in the top 10 positions. Notably, most of the top-ranked miRNAs enriched in giant panda breast milk are also abundant in human^18^, porcine^28^ and bovine^29^ breast milk and have been associated with developmental processes and immune responses based on the annotation of the Pathway Central database (SABiosciences, MD, USA). For example, members of the let-7 family that are highly expressed in various species, including mammals, flies, worms, and plants^30^, are well-known regulators of development, cellular basal metabolism, and innate immune responses^31^. miR-148a regulates the innate response and antigen presentation of Toll-like receptor (TLR)-triggered dendritic cells by targeting CaMKIIα^32^. MiR-92a, member of the miR-17-92 cluster, negatively regulates TLR-triggered inflammatory responses in macrophages by targeting MKK4 kinase^33^. MiR-30a promotes B cell hyperactivity in patients with systemic lupus erythematosus by direct interactions with Lyn^34^. MiR-30d enhances invasion and immunosuppression during metastasis by targeting GalNAc transferases^35^. MiR-200a-3p is associated with various immunological diseases, such as Hodgkin lymphoma^36^ and oral cancers^37^. Therefore, the high abundance of the above miRNAs in the milk of panda can affect the physiology, and particularly the immune system, of the newborn cubs. To induce an effect on the immune system of the cubs, the miRNA need to be horizontally transferred (or intestinally absorbed).

### Distinct miRNA expression patterns during different lactation stages

**Figure 3:**
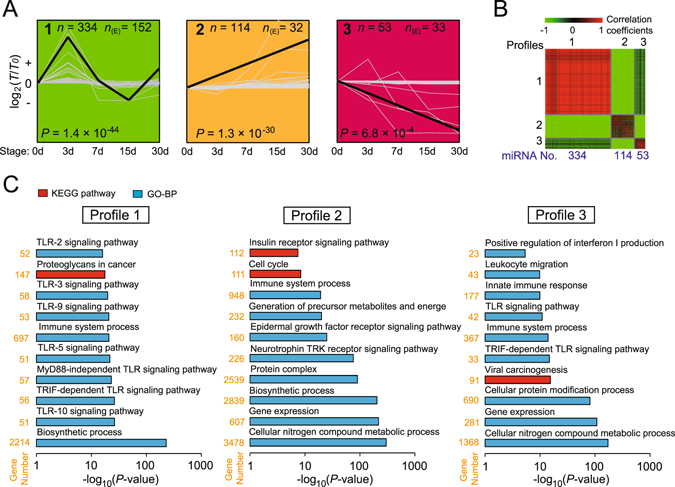
Temporal expression of exosomal miRNAs in giant panda breast milk. (**A**) Three profiles that have significantly more miRNAs assigned under the true ordering of time points compared to the average number assigned to the model profile in the permutation runs (non-significant profiles are not shown). The upper left represents the serial number of the cluster, the lower left represents the *P* value (Fisher’s exact test), *n* represents the number of genes assigned, and *n*_(E)_ represents the number of genes expected. All expressions were log_2_-transformed. (**B**) Heat map of significant profiles. Gene pairs that are positively or negatively correlated are shown in red or green, respectively. (**C**) GO categories and pathways enriched for target genes of significant model profiles. The *P* value, indicating the significance of the comparison, was calculated using the *Benjamini*-corrected modified Fisher’s exact test.

To visually illustrate the expression patterns of the miRNAs during different development stages, a short time-series expression miner (STEM) analysis was performed. The 1,194 unique miRNAs were clustered into 20 distinct expression patterns, of which 501 miRNAs were significantly over-enriched in three model profiles (Fig. 3A and Supplementary Table S3). There was a significantly larger-than-expected number of genes assigned to the model profile in the permutation runs (*P* < 0.01). The expression levels of miRNAs within a single profile are strongly correlated, whereas miRNAs belonging to different profiles generally showed no significant co-expression pattern (Fig. 3B), further confirming the results of STEM. Profile 1 comprised 334 unique miRNAs, whose expression gradually increased from 0 to 3 d, peaking on day 3 and subsequently decreasing to 15 d. Then, it increased again to 30 d. Profiles 2 and 3 comprised 114 and 54 unique miRNAs, respectively. The expression levels of miRNAs in profile 2 persistently increased following delivery, while the expression levels of profile 3 miRNAs decreased in this timeframe. These three profiles may indicate the different roles of corresponding miRNAs; therefore, we performed target prediction and functional annotation analyses of the conserved miRNAs in these three profiles using DIANA-mirPath^38^ (see Methods for details). As shown in Fig. 3C, the target genes of conserved miRNAs that were highly expressed in the giant panda colostrum (0, 3 and 7 d; profiles 1 and 3) were primarily involved in immune and biosynthetic processes, including the “TLR signaling pathway,” “immune system process,” “proteoglycans in cancer,” “innate immune response,” “viral carcinogenesis” and “cellular nitrogen compound metabolic process”. Recent reports have indicated that secreted exosomal miRNAs were enriched in the breast milk of various mammals, such as humans^8^, cows^19^, pigs^39^ and rats^40^, and function as potential gene silencing signals between mothers and infants. The enrichment of these immune-related miRNAs in the giant panda colostrum may also imply their vital regulatory function in the neonatal immune system similar to other colostral immunological components. The colostral immunological components, such as immunoglobulin, cytokines, and leukocytes, that are transferred into the peripheral blood of the neonate play an important role in the development of the neonatal immune system^41^.

In contrast, the target genes of highly expressed miRNAs in mature milk (15 and 30 d; profiles 2) were primarily related to biosynthetic process, cell differentiation and gene expression, which are actively involved in body growth and development, such as “cell cycle,” “generation of precursor of metabolites and energy,” “cellular nitrogen compound metabolic process,” and “protein complex”, and may be essential for the rapid development and growth of the premature newborn cubs. For example, miR-450a-5p promotes adipogenic differentiation by targeting *WISP2* ^42^. MiR-34a promotes neural progenitor cell differentiation by targeting *Numblike*. MiR*-*135b*-*5p^43^ and miR-664b-3p^44^ can promote cell proliferation by targeting *MEF2C* and *PLP2*, respectively. MiR-183-5p^45^ and miR-200b-3p^46^ can positively regulate the development of inner ear. From 140 g (newborn) to 40 kg (weaning) of body weight, the growth and development of the giant panda primarily relies on the postnatal breast-feeding milk until weaning at 1–1.5 years old^3^ based on many energy, protein and regulatory factors. This dependence may be the primary reason that miRNAs that play vital roles in growth and development are highly expressed in mature milk.

### Resistance of milk exosomal miRNAs to the simulated gastrointestinal environment

**Figure 4:**
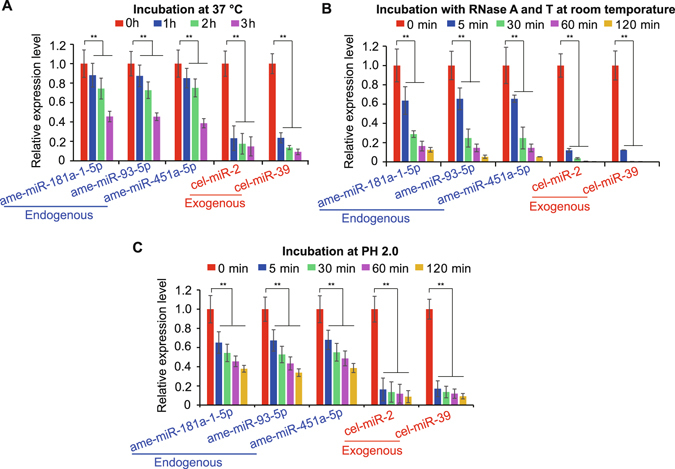
Stability of exosomal miRNAs in simulated harsh gastrointestinal conditions. The expression changes of two spiked-in synthetic *C*. *elegans* miRNAs and three endogenous miRNAs across various harsh conditions. Total RNA was extracted and subsequently analyzed by q-PCR. Breast milk was (**A**) incubated at 37 °C for 1, 2 or 3 hours, (**B**) subjected to RNase A and T for 5, 30, 60 or 120 min at 37 °C, or (**C**) treated at pH 2.0 at 37 °C for 5, 30, 60 or 120 min. Three independent experiments performed in triplicate and all data are expressed as mean ± SD. **P < 0.01.

The resistance to harsh conditions of the gastrointestinal environment is a precondition of maternal exosomal miRNAs absorbed by the infant intestinal wall. To investigate the stability of exosomal miRNAs of giant panda breast milk, three endogenous and two exogenous miRNAs were randomly selected and subjected to a simulated gastrointestinal environment. Two exogenous synthetic worm-specific miRNAs (cel-miR-2 and cel-miR-93) were sharply degraded; however, the endogenous milk-derived miRNAs exhibited resistance to degradation when the milk was subjected to prolonged incubation at 37 °C, low pH or even treatment with high concentrations of exogenous RNase (Fig. 4). These results suggest that milk-derived miRNAs exist in a remarkably stable form, protected from endogenous RNase activity across various harsh conditions. This result is consistent with previous studies on blood^47^ and human^8^ and bovine^19^ breast milk. The remarkable stability of the endogenous milk-derived miRNAs provides a basic condition for the dietary intake of maternal miRNAs by infants.

### Exogenous plant miRNAs were detected in the milk exosomes of the giant panda

**Figure 5:**
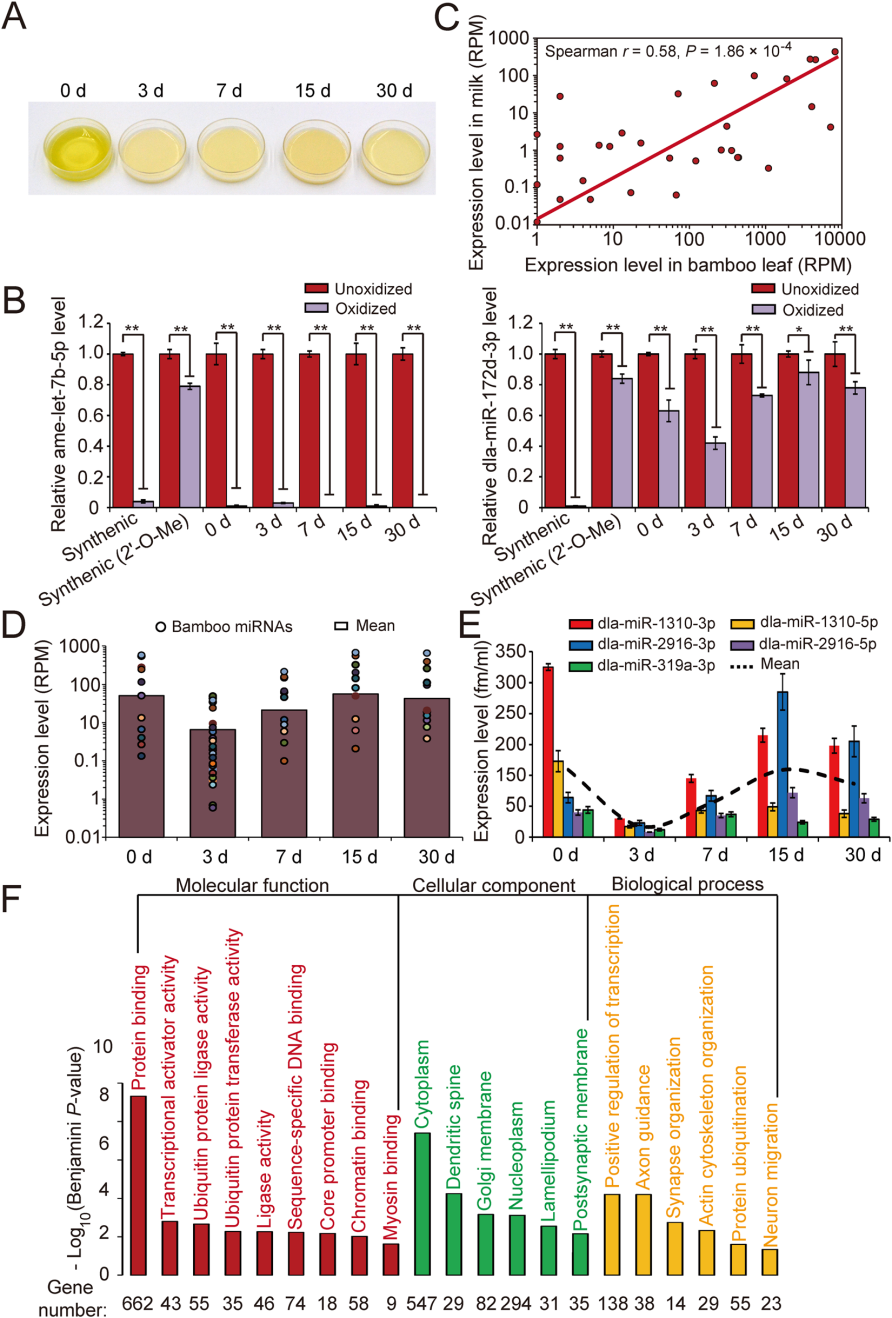
Bamboo miRNAs are present in the breast milk of giant panda. (**A**) The color of the breast milk of the giant panda during distinct lactation periods. (**B**) Equal amounts of synthetic plant (dla-miR-172d-3p) and giant panda (ame-let-7b-5p) small RNAs (with or without 2′-O-methylated 3′ ends) were treated with/without sodium periodate. Subsequently, the endogenous and plant miRNAs levels were detected via a qRT-PCR assay. (**C**) The expression correlation of bamboo miRNAs in bamboo leaves and giant panda breast milk. (**D**) The expression levels of plant miRNAs in the breast milk of the giant panda during distinct lactation periods, detected using small RNA-seq and (**E**) stem-loop qRT-PCR. (**F**) The functional enrichment analysis of exosome-loaded exogenous bamboo miRNAs. All data are expressed as mean ± SD. *P < 0.05, **P < 0.01.

Interestingly, in the present study, we observed that the breast milk of the giant panda was colored yellowish green in 0 day colostrum and faded to milk white in 3 days; then, it gradually turned back to light yellowish green at 7 days (Fig. 5A), indicating that there are dynamic plant ingredients in the milk. Considering that giant pandas live primarily on bamboo and recent findings that plant miRNAs exist in mammalian body fluids^48^, we proposed that food-derived plant miRNAs also exist in breast milk. To test this hypothesis, bamboo miRNA species and their abundance were detected by mapping the small RNA-seq data to the bamboo genome and known plant precursor miRNAs in the miRBase. In total, 37 mature bamboo miRNAs corresponding to 27 precursor miRNAs were observed in the giant panda breast milk (Supplementary Table S4). To determine whether the bamboo miRNAs identified in giant panda breast milk are genuine plant miRNAs, we treated the total small RNAs isolated from giant panda milk with sodium periodate (oxidizing agent)^49^. Consequently, compared with the complete degradation of endogenous giant panda miRNAs (ame-let-7b-5p, ame-miR-181 and ame-miR-451), the bamboo-derived miRNAs (dla-miR-172d-3p, dla-miR-535-5p, dla-miR-1310-3p, dla-miR-1561-5p and dla-miR-2916-5p) exhibited similar resistance as synthetic miRNAs with 2′-Omethylated 3′ ends, highlighting the identity of bona fide plant miRNAs (Fig. 5B and Supplementary Fig. S4). Notably, the abundance levels of exogenous bamboo miRNAs in giant panda milk were significantly correlated with their intrinsic expression levels in bamboo leaf (Spearman’s *r* = 0.58, *P* = 1.86 × 10^−4^) (Fig. 5C, Supplementary Table 5) detected using a small RNA-seq approach. The moderate rather than high correlation may result from the different biodegradation degrees of bamboo miRNAs in long gastrointestinal tract before being absorbed, and from the selective packaging of intracellular miRNAs into exosomes/microparticles^50–56^. In addition, both small RNA-seq and stem-loop qRT-PCR results indicated that the abundance of exogenous bamboo miRNAs changed with the increasing greenness of giant panda milk (Fig. 5D,E), which may be highly associated with the stress fasting of the giant panda within the first three days after delivery^57^. This result emphasizes the potential oral intake of exogenous plant miRNAs from food sources in the giant panda. Functional enrichment analyses of the target genes indicated that the top ten exogenous plant miRNAs were primarily involved in basic cell metabolism, such as “protein binding,” “transcriptional activator activity,” “ubiquitin protein transferase activity” and “chromatin binding,” and neurodevelopment, such as “synapse organization,” “neuron migration” and “axon guidance” (Fig. 5F and Supplementary Table S6). These results indicated the potential role of exosome-loaded exogenous bamboo miRNAs in the postnatal development of giant panda cubs.

## Materials and Methods

### Animal ethics statement

All breast milk samples were collected from captive-bred giant pandas at the Bifengxia Chinese conservation and research center for the giant panda in the Sichuan province of China. Giant pandas were fed a diet of local bamboo supplemented with bread, high-fiber biscuits, carrots and apples. All animals used in this study were farmed according to the Technical Regulation of Husbandry and Management of the Giant Panda (Chinese National Standard, LY/T 2015-2012) and approved by the Institutional Animal Care and Use Committee in the College of Animal Science and Technology, Sichuan Agricultural University, Sichuan, China. All methods were carried out in accordance with the approved guidelines and regulations.

### Preparation of experimental animals and samples

Three female giant pandas were sampled over multiple lactations, including 0, 3, 7, 15 and 30 days after delivery. Breast milk samples (5–10 ml) were collected from each stage, and a portion of each milk sample was stored at 4 °C for atomic force microscope (AFM) analysis. The remaining portion of each milk sample was centrifuged at 2500 × *g* for 10 min, twice, to remove cells and fat globules. The supernatant was centrifuged at 12,000 × *g* for 30 min at 4 °C to remove any cellular debris. The supernatants were then frozen in liquid nitrogen and subsequently stored at −80 °C until exosome enrichment and RNA extraction.

### Atomic force microscope (AFM) assay

The breast milk exosomes for AFM analysis were prepared as described by Gu *et al*.^28^, with minor modifications. Briefly, 5 ml of breast milk was centrifuged at 2500 × *g* for 10 min, twice, to remove cells and fat globules. The supernatant was centrifuged at 12,000 × *g* for 30 min at 4 °C to remove any cellular debris. The supernatants were subsequently centrifuged at 120,000 × *g* for 4 hours, and the pellet was re-suspended in 10 ml of phosphate buffered saline (PBS). The exosome solutions were diluted 1:500 in deionized water and adsorbed to freshly cleaved mica sheets for 10 min. The redundant solution was removed by careful absorption with a filter paper, and the mica was further dried prior to detection. Surface morphology was examined under an AFM (Asylum Research MFP-3D-Bio, Digital Instruments Inc., Santa Barbara, CA) using identical conditions. The size of the exosomes was determined from a line profile of the AFM image.

### Enrichment of exosomal RNA from breast milk

RNA in the breast milk exosomes was isolated as previously described^28^. In brief, raw milk (1.5 ml) was centrifuged at 2,000 × *g* for 10 min to remove fat globules. The supernatant was centrifuged at 12,000 × *g* for 30 min and further filtered through a 0.45 mm PVDF filter to eliminate cells and cellular debris. Approximately 1 ml of the supernatant was mixed with 500 ml of ExoQuick Exosome Precipitation Solution (SBI, CA, USA) and incubated at 4 °C for 12 hours. The ExoQuick/supernatant mixture was centrifuged at 1,500 × g for 30 min to obtain a beige exosome pellet, which was then re-suspended in 250 ml nuclease free water. Total RNA from the exosomes was extracted using TRIzol-LS (Invitrogen, CA, USA), following the manufacturer’s instructions. Small RNAs were analyzed with the Agilent Bioanalyzer 2100 and the RNA 6000 Nano LabChip Kit (Agilent, CA, USA).

### Western blot analysis

Thirty micrograms of protein was separated on an 8% SDSPAGE gel and transferred to polyvinylidenedifluoride membranes (Millipore). Membranes were blocked and then incubated for 2 hours with either rabbit anti-alpha Tubulin (1:1000, Abcam), mouse anti-CD63 (1:1000, Abcam) or mouse anti-TSG101 (1:200, Abcam). Horseradish peroxidase-conjugated anti-mouse or anti-rabbit IgG was used as a secondary antibody (diluted 1:5,000 in TBST). Bands were scanned using a densitometer (GS-700; Bio-Rad Laboratories).

### Small RNA sequencing

Total RNAs isolated from three individuals at four lactation stages (0, 7, 15 and 30 days after delivery) were pooled at equal quantities for library construction of each stage. To evaluate the variation among individuals, the total RNA from the milk-derived exosmes of three giant pandas at 3 days were used separately for library construction. In total, seven libraries were subjected to single-end sequencing of 36 nt reads using an Illumina Genome Analyzer II. The bioinformatics pipeline for miRNA discovery and profiling was used as previously described, with some improvements^58^. All reads were counted, and the identical reads were combined into a single type. The raw reads were subjected to a series of additional strict filters (such as the digital filters of base-call quality, read length, and sequence comparison) with acceptance criteria derived from the statistics of mammalian and plant miRNAs in miRBase 21.0. The reads originated from known RNAs of the giant panda and bamboo (i.e., mRNA; rRNA, tRNA, snRNA, and snoRNA), and repetitive sequence elements were also filtered. The raw reads were passed through a series of filters and mapped to all known pre-miRNAs of mammals and plants registered in miRBase 21.0, and the Giant Panda (*Ailuropoda melanoleuca*, AilMel1.0) and bamboo (http://www.ncgr.ac.cn/bamboo) genomes using NCBI Local BLAST. All candidates who did not map to any known pre-miRNAs but map to genome were considered as potential novel miRNAs. To further identify these potential novel miRNA candidates, MIREAP software^59^ was used to predict novel miRNA by exploring the secondary structure, the Dicer cleavage site and the minimum free energy of the annotated small RNAs.

### Short time-series expression miner (STEM) analysis

STEM analysis was used to visualize expression patterns for unique miRNAs. Each miRNA was assigned to the model profile most closely matched to its time series based on the correlation coefficient. The number of miRNAs assigned to each model profile was subsequently computed. The number of miRNAs assigned to a profile was estimated by randomly permuting the original time point values, renormalizing the miRNA expression values, assigning the miRNAs to their most closely matching model profiles, and repeating this process for a large number of permutations. The average number of miRNAs assigned to a model profile over all permutations was used as the estimate of the expected number of miRNAs assigned to the profile. The significance of the number of miRNAs assigned to each profile compared with the expected number was also computed^60^.

### Databases of known signaling pathways

The pathways knowledge base developed by SABiosciences (SABiosciences, MD, USA) (http://www.sabiosciences.com/pathwaycentral.php) was used to annotate the miRNAs of interest in known pathways, as described previously^61^.

### Prediction and functional annotation of miRNA target genes

DIANA-mirPath^38^, a web-based computational tool, was employed to identify the target genes of giant panda miRNAs and the molecular Gene Ontology (GO) and pathways potentially altered by the expression of multiple miRNAs (http://www.microrna.gr/miRPathv3). TargetScan human 5.2^62^ was used for the target prediction of exogenous bamboo miRNAs based on seed sequences. Subsequently, the Gene Ontology and Kyoto Encyclopedia of Genes and Genomes (KEGG) pathway terms enriched in the predicted target genes were determined using Database for Annotation, Visualization and Integrated Discovery (DAVID) bioinformatics resources^63^. The predictions were made according to the interactions of human mRNA-miRNA due to the absence of giant panda and bamboo miRNAs in the current version of the above-mentioned algorithm.

### qRT-PCR and clone sequencing validation of conserved and novel miRNAs

The changes in abundance of seven conserved and eight novel giant panda miRNAs over five lactation stages were determined by an EvaGreen-based qRT-PCR approach using a High-Specificity miRNA qRT-PCR Detection Kit (Stratagene, La Jolla, USA) on the CFX96^TM^ Real-Time PCR Detection System (Bio-Rad, CA, USA). The U6 snRNA, 5 S rRNA, and Met-tRNA were simultaneously used as endogenous control genes. All measurements contained a negative control (no cDNA template), and each RNA sample was analyzed in triplicate. Relative expression levels of objective miRNAs were calculated using the 2^−ΔΔCt^ method. To ensure the accuracy of the 2^−ΔΔCt^ results, we have carried out the optimization of annealing temperature by thermal gradient and evaluation of amplification efficiency in CFX96^TM^ Real-Time PCR Detection System (Bio-Rad, CA, USA), and all wells used in the subsequent analysis were met 0.95 < AE < 1.05, including the target genes and endogenous controls.

The PCR products of novel miRNAs were then cloned into plasmid using the TOPO TA Cloning Kit (Invitrogen, Paisley, UK), and subsequently sequenced. Stem-loop qRT-PCR was used to evaluate the exogenous plant miRNA level by Bulge-LoopTM miRNA qRT-PCR Starter Kit (RIBOBIO, China) according to manufacturer’s instructions, and the transcriptional and qPCR primers were also synthesized by RIBOBIO (Guagnzhou, China) and shown in Supplementary Table S7. All measurements contained a negative control (no cDNA template), and each RNA sample was analyzed in triplicate.

### Simulated gastrointestinal harsh treatments of miRNAs

Two exogenous miRNAs (cel-miR-2-3p, and cel-miR-39-5p of *C*. *elegans*), which have no sequence similarity to the giant panda miRNAs, were chemically synthesized. To determine the resistance and stability of endogenous exosomal miRNAs in the giant panda milk, exogenous miRNAs were added directly to raw milk, which were then subjected to three types of treatments: (A) incubation at 3 7 °C for 1, 2, or 3 hours; (B) treatment in pH 2.0 solution for 5, 30, 60, or 120 minutes; (C) treatment with RNase A (0.16 mg mL^−1^) and RNase T1 (0.4 U mL^−1^) (Fermentas, Shenzhen, China) for 5, 30, 60 or 120 minutes at 37 °C. The treated milk samples was centrifuged at 2500 × *g* for 10 min, twice, to remove cells and fat globules. The supernatant was centrifuged at 12,000 × *g* for 30 min at 4 °C to remove any cellular debris. The supernatants were then used for total RNA extraction using TRIzol-LS (Invitrogen, CA, USA).

### Oxidation of small RNAs with periodate

Total RNA was extracted from giant panda milk using Trizol LS Reagent (Invitrogen), and synthetic ame-let-7b-5p, ame-miR-181, ame-miR-451, dla-miR-172d-3p, dla-miR-535-5p, dla-miR-1310-3p, dla-miR-1561-5p and dla-miR-2916-5p (with or without 2′-O-methyl) were obtained from RIBOBIO (Guangzhou,China). A 10 μl aliquot of total RNA or synthetic miRNA was mixed with 10 μl of NaIO_4_ (0.25 M) and 80 μl of RNase-free water, and incubated at 0 °C for 40 min in the dark. Approximately 10 μl of NaIO_4_ was replaced by 10 μl of RNase-free water in the unoxidized group. Next, the RNA was precipitated using 80% ethanol, aired dried, dissolved in RNase-free water, and subsequently assayed using stem-loop qRT-PCR according to the procedure described above.

### Statistical analysis

All data from qRT-PCR are expressed as mean ± SD. The statistical analysis was performed using SPSS 19.0 software. Equality of group variances were assessed by Kolmogorov-Smirnov test. Statistical significance was calculated by one way analysis of variance (ANOVA) with Tukey’s post-hoc test for multiple groups or Student’s t-test for comparisons of two groups. *P* < 0.05 was considered statistically significant (*P < 0.05; **P < 0.01). Spearman’s correlations were used to examine the relationship between miRNA expression levels detected by small RNA-sequencing and qRT-PCR, and between bamboo miRNA expression levels presented in bamboo leaf and milk-derived exosome.

## Conclusions

The present study presents a comprehensive survey of the exosomal miRNA transcriptome in giant panda breast milk during five lactation stages, shedding new light on the gene regulation network during the development of newborn cubs. Immune- and development-related endogenous miRNAs were observed to be enriched in the exosomes of giant panda breast milk, which together with evidence that these exosomal miRNAs are resistant to harsh gastrointestinal conditions, suggested that these exosomal miRNAs may be genetic material transferable from mother to infant and are essential for the development of newborn cubs. In addition, these findings support the viewpoint that food-derived exogenous plant miRNAs can be taken up and presented in mammalian breast milk.

## Electronic supplementary material

Supplementary information

Table S1

Table S2

Table S3

Table S4

Table S5

Table S6

Table S7
