# Supplementary material for: Exosomal microRNAs in giant panda (Ailuropoda melanoleuca) breast milk: potential maternal regulators for the development of newborn cubs
Source: Sci Rep. 2017 Jun 14;7:3507. doi: 10.1038/s41598-017-03707-8 (PMC5471263; doi:10.1038/s41598-017-03707-8)
Supplement: Supplementary file 1 — Supplementary information [file 41598_2017_3707_MOESM1_ESM.pdf]

Supplementary information

Exosomal microRNAs in giant panda *(Ailuropoda melanoleuca*) breast milk: potential maternal regulators for the development of newborn cubs

Jideng Ma[1†], Chengdong Wang[1,2†], Keren Long[1†], Hemin Zhang[1,2†]*, Jinwei Zhang[1], Long Jin[1], Qianzi Tang[1], Anan Jiang[1], Xun Wang[1], Shilin Tian[1,3], Li Chen[1], Dafang He[1], Desheng Li[2], Shan Huang[2], Zhi Jiang[3]*, Mingzhou Li[1]*

[1] Institute of Animal Genetics & Breeding, College of Animal Science & Technology, Sichuan Agricultural University, Wen'jiang 611130, China;

[2] China Conservation and Research Center for the Giant Panda, Wolong, Sichuan, China

[3] Novogene Bioinformatics Institute, Beijing 100089, China;

*Correspondence should be addressed to H.Z (Email: wolong_zhm@126.com) , Z.J (Email: jiangzhi@novogene.com) or M.L(Email: mingzhou.li@sicau.edu.cn)

[†] These authors contributed equally to this work.

Supplementary Figure 1. RNA from breast milk exosomes was detected using Agilent Bioanalyzer 2100.

Supplementary Figure 2. Identification of endogenous exosome miRNAs from giant panda breast milk. **(A)** Classification of small RNAs presented in the exosomes of giant panda milk. **(B)** Length distribution of identified miRNAs in all seven small RNA-seq libraries. **(C)** The high correlation of miRNA expression between three biological replicates.

Supplementary Figure 3. Validation of small RNA sequencing results. **(A)** Alignment of small RNA sequencing and clone sequencing results for novel miRNAs discovered in the giant panda. **(B)** Validation of the expression patterns of conserved miRNAs using a qRT-PCR approach.

Supplementary Figure 4. The resistance of endogenous giant panda miRNAs and dietary exogenous plant miRNAs to periodate oxidation. Equal amounts of synthetic plant (dla-miR-535-5p, dla-miR-1561-5p, dla-miR-1310-3p and dla-miR-2916-5p) and giant panda (ame-miR-181-5p and ame-miR-451-5p) small RNAs (with or without 2'-O-methylated 3' ends) were treated with/without sodium periodate. After the reactions, the endogenous and plant miRNAs levels were detected using a qRT-PCR assay. Three independent experiments performed in triplicate and all data are expressed as mean ± SD. $^*P < 0.05$, $^{**}P < 0.01$.

Supplementary Table 1. Conserved and novel giant panda miRNAs identified in this study.

Supplementary Table 2. The expression of giant panda unique miRNAs.

Supplementary Table 3. The abundance of miRNA members of significantly over-enriched model profiles.

Supplementary Table 4. The expression of exogenous bamboo miRNAs presented in giant panda milk exosomes.

Supplementary Table 5. The expression of bamboo miRNAs identified in bamboo leaf.

Supplementary Table 6. The functional prediction for target genes of exogenous bamboo miRNAs presented in giant panda milk exosomes.

Supplementary Table 7. Primer sequences of the q-PCR experiments.

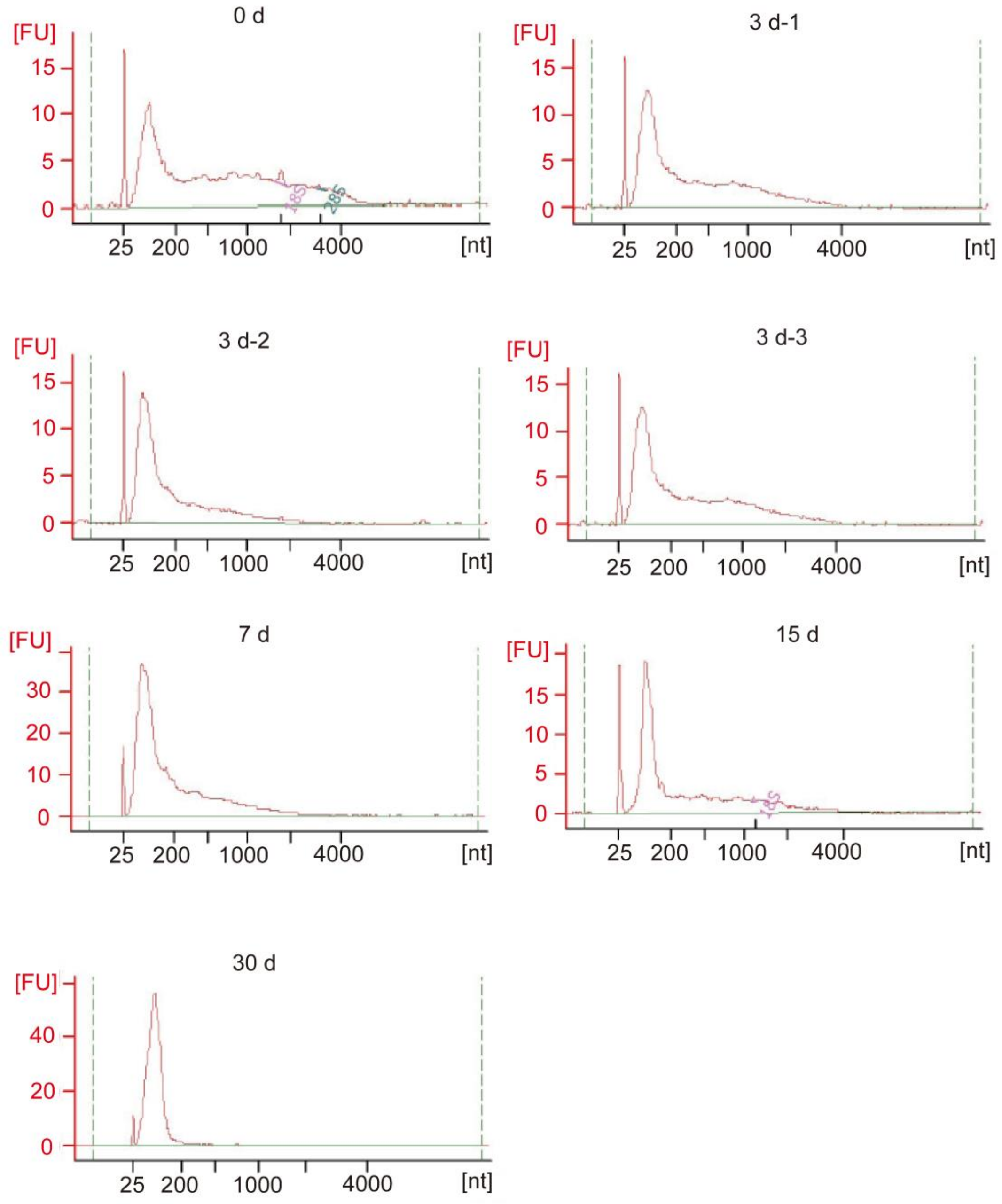

Supplementary Figure 1. RNA from breast milk exosomes was detected using Agilent Bioanalyzer 2100.

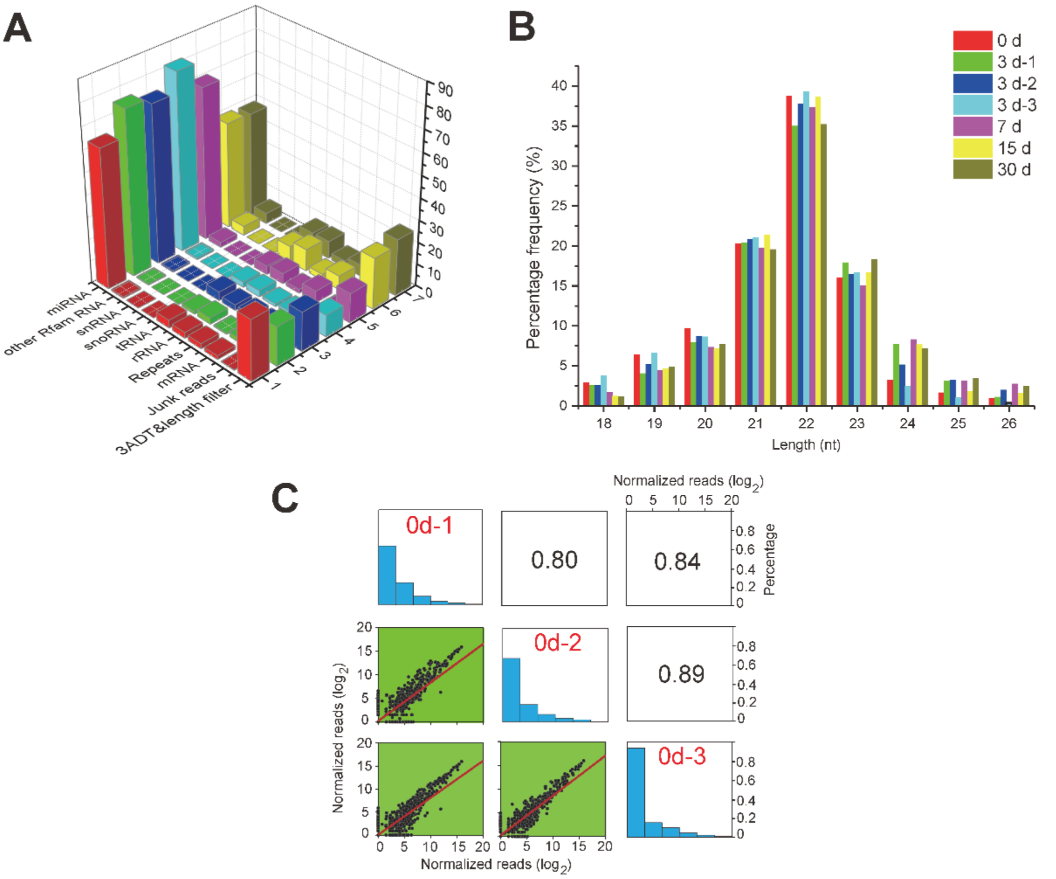

Supplementary Figure 2. Identification of endogenous exosome miRNAs from giant panda breast milk. **(A)** Classification of small RNAs presented in the exosomes of giant panda milk. **(B)** Length distribution of identified miRNAs in all seven small RNA-seq libraries. **(C)** The high correlation of miRNA expression between three biological replicates.

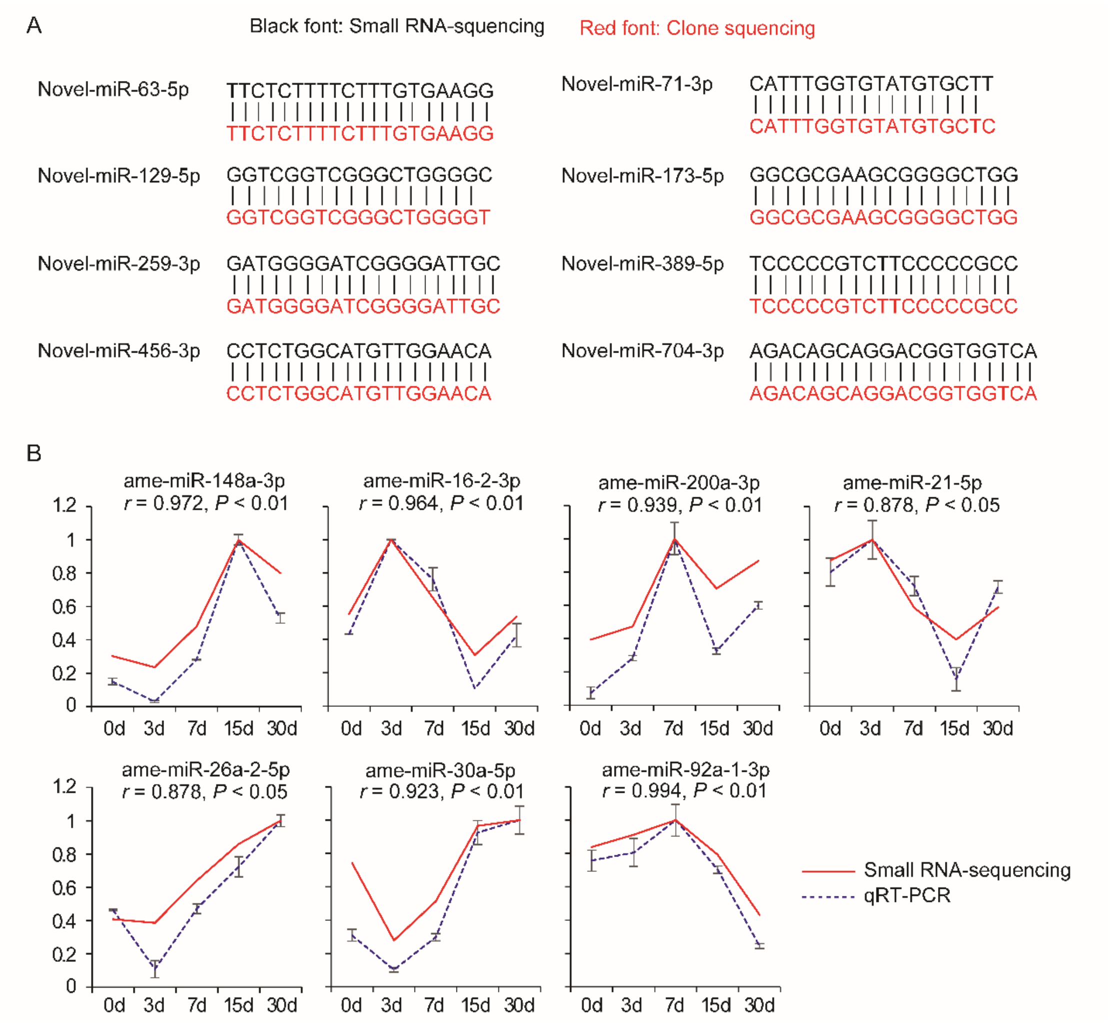

Supplementary Figure 3. Validation of small RNA sequencing results. **(A)** Alignment of small RNA sequencing and clone sequencing results for novel miRNAs discovered in the giant panda. **(B)** Validation of the expression patterns of conserved miRNAs using a qRT-PCR approach.

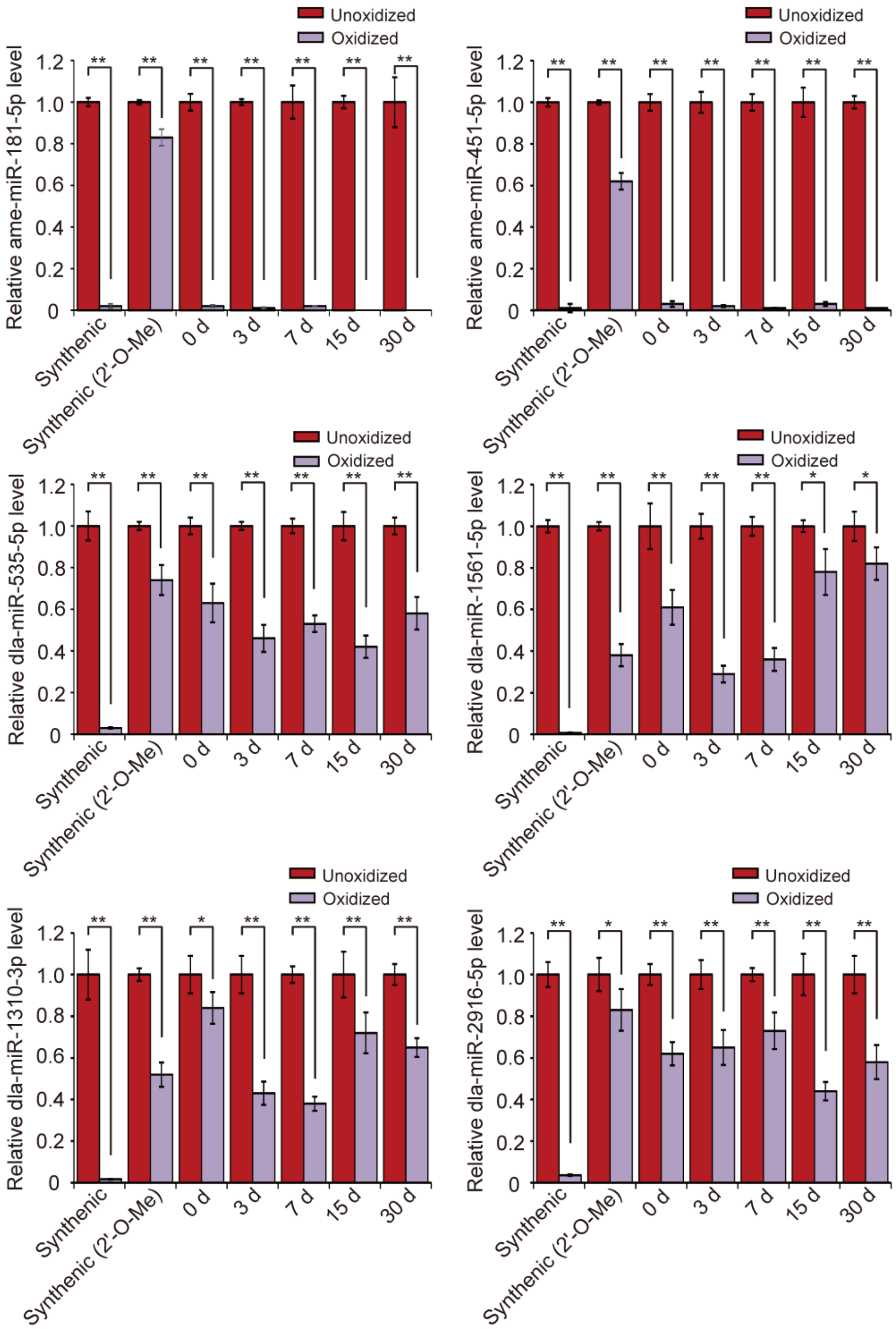

Supplementary Figure 4. The resistance of endogenous giant panda miRNAs and dietary exogenous plant miRNAs to periodate oxidation. Equal amounts of synthetic plant (dla-miR-535-5p, dla-miR-1561-5p, dla-miR-1310-3p and dla-miR-2916-5p) and giant panda (ame-miR-181-5p and ame-miR-451-5p)

small RNAs (with or without 2'-O-methylated 3' ends) were treated with/without sodium periodate. After the reactions, the endogenous and plant miRNAs levels were detected using a qRT-PCR assay. Three independent experiments performed in triplicate and all data are expressed as mean ± SD. *P <0.05, **P < 0.01.
